# The Big Five Personality Domains and Their Facets: Empirical Relations to Problematic Use of Video Games, Social Media and Alcohol

**DOI:** 10.3390/bs13060444

**Published:** 2023-05-24

**Authors:** Lutz Wartberg, Katrin Potzel, Carolin Spindler, Rudolf Kammerl

**Affiliations:** 1 Department of Psychology, Faculty of Human Sciences, MSH Medical School Hamburg, 20457 Hamburg, Germany; 2 Department of Education, Chair for Pedagogy with a Focus on Media Education, Friedrich-Alexander-University Erlangen-Nuremberg, 90478 Nuremberg, Germany

**Keywords:** gaming disorder, internet addiction, social media addiction, binge drinking, adolescent, openness to experience, conscientiousness, extraversion, agreeableness, neuroticism

## Abstract

Relatively common behavioral patterns in adolescence are problematic use of video games (PG), social media (PSMU) or alcohol (PAU). According to theoretical models, personality traits are relevant for Internet-related problematic behaviors. In the present study, associations of the Big Five personality domains and their 15 facets with PG, PSMU and PAU were compared for the first time. Therefore, 492 adolescents (mean age: 16.83 years) were examined with the established Big Five Inventory-2 as well as other standardized questionnaires on PG, PSMU and PAU. For statistical evaluation, correlation analyses were used as bivariate procedures and multiple regression analyses as multivariable procedures. At the personality domain level, consistently in bivariate and multivariate analyses, statistically significant associations between higher Negative Emotionality (Neuroticism) and PG, PSMU and PAU as well as between lower Open-Mindedness and PG and PAU were observed. At the level of facets, higher Anxiety (facet of Negative Emotionality) was related to PG and PSMU as well as lower Aesthetic Sensitivity (facet of Open-Mindedness) and lower Productiveness (facet of Conscientiousness) to PG. Considering the overlap of 95% confidence intervals, very comparable patterns of associations between PG, PSMU and PAU and the Big Five and their facets were observed in adolescence (indicating similarities in etiology).

## 1. Introduction

The use of video games is one of the most popular leisure activities both internationally and in Germany, with more than three billion gamers worldwide in 2022 [1]. In Germany, where the present survey was conducted, the estimated number of gamers is 34.3 million [2], and in the age group of 16 to 29 years, for example, almost nine out of ten persons (88%) use video games [3]. According to the results of a representative sample of German 12- to 19-year-olds, only 6% of this age group never play video games, while 76% play several times a week or every day [4].

For most children and adolescents worldwide, video games are an enjoyable leisure activity. However, according to the available empirical findings, a subgroup of minors develops problematic patterns of gaming use. In Germany, in a representative sample of 12- to 17-year-olds a prevalence estimate of 3.5% for problematic use of video games was observed [5]. With the inclusion of the (research) diagnosis Internet Gaming Disorder into the DSM-5 [6] and the diagnosis Gaming Disorder into the ICD-11 (e.g., [7]), respectively, problematic gaming has meanwhile been incorporated in the most important international classification systems for mental disorders.

In the ICD-11, in addition to the “Disorders due to substance” (substance-related disorders), there is for the first time a category “Disorders due to addictive behaviours” (non-substance-related disorders), in which Gaming Disorder is also classified. In the category “Disorders due to addictive behaviours”, Gambling and Gaming Disorder are directly mentioned (but other behavioral patterns such as problematic use of social media are not). However, problematic use of social media is not a rare phenomenon in youth [5]. To measure this phenomenon, the DSM-5 and ICD-11 criteria for video games were adapted for problematic use of social media and standardized instruments were developed on this basis (e.g., [8]). Based on one of these instruments (the Social Media Disorder Scale, [8]), a prevalence estimate was made in a representative sample of German 12- to 17-year-olds, and a problematic use of social media was observed in 2.6% of children and adolescents [5]. The most common substance-related problematic behavior among minors in Germany is problematic alcohol use, and in a representative sample, it showed up in 5.0% of 12- to 17-year-olds [9]. In summary, a substantial proportion of German children and adolescents reported either problematic use of digital media applications (such as video games or social media) or problematic use of psychotropic substances (alcohol being the most common).

An important theoretical approach to the development of Internet-related disorders and thus also problematic use of video games or social media is the Interaction of Person-Affect-Cognition-Execution (I-PACE) model [10]. In this model, the interaction of characteristics of the person with her or his affects, cognitions and executive functions is described. For the development of Internet-related disorders, personality traits are explicitly named in addition to psychopathological stress, social cognitions, motives for use and biopsychological constitution (e.g., genetic factors).

There are various approaches to survey personality traits (which are the focus of this evaluation). The best-known and most frequently used approach in personality research for describing basic personality traits is the five-factor-model (so-called Big Five, [11]). The Big Five could be empirically confirmed cross-culturally and can be abbreviated with the acronym OCEAN. OCEAN stands for the five personality dimensions: (I) Openness to experience, (II) Conscientiousness, (III) Extraversion, (IV) Agreeableness and (V) Neuroticism. According to Groß and Kohlmann [12], the Big Five can be briefly described by: (I) fancifully, active imagination (Openness to experience), (II) thoroughness vs. convenience (Conscientiousness), (III) sociable vs. reserved (Extraversion), (IV) tendency to criticize others vs. trust others (Agreeableness) and (V) nervous, insecure vs. relaxed (Neuroticism). There are different standardized instruments for surveying the five-factor-model or the Big Five. A distinction can be made between questionnaires that only collect the Big Five personality domains (such as the NEO-Five-Factor Inventory, NEO-FFI, [13]) and those that allow an additional differentiation into facets (examples are the Revised NEO Personality Inventory, NEO-PI-R [14], which can be used to describe a total of 30 facets of the Big Five, or the Big Five Inventory 2, BFI-2 [15], which can be utilized to examine 15 facets). The facets make a further differentiation of the Big Five possible, which can definitely provide additional insight [15]. For example, regarding the personality domain Conscientiousness, with the BFI-2 three facets of it (Organization, Productiveness and Responsibility) are distinguished.

In several cross-sectional studies associations between the personality domains of the Big Five [11] and problematic video game use in youth were investigated. Vollmer et al. [16] surveyed “computer game addiction” in Turkish children and adolescents (mean age: 12.89 years) and found relations to lower Extraversion, lower Agreeableness, and higher Neuroticism. Wang et al. [17] examined “gaming addiction” in Chinese adolescents (average age: 15.03 years) and reported associations with lower Conscientiousness and lower Openness to experience. López-Fernández et al. [18] assessed “disordered gaming” in Spanish adolescents (mean age: 14.29 years) and observed relationships with lower Conscientiousness and lower Agreeableness. López-Fernández et al. [19] reported direct associations between disordered gaming and lower Conscientiousness as well as higher Neuroticism for Spanish adolescents (average age: 14.97 years) who used video games at least once a week. Sánchez-Llorens et al. [20] studied “video game addiction” in Spanish adolescents (mean age: 14.85 years) and obtained a statistically significant correlation to lower Agreeableness. Compared to participants with video game addiction, a healthy control group showed higher scores in the personality domains of Conscientiousness and Agreeableness [20]. In addition, the very first findings from longitudinal studies are now available. Ok [21] investigated South Korean adolescents (average age: 15.07 years at the first time of measurement) and identified lower Conscientiousness and higher Extraversion as predictors of “problematic game use”. Marrero et al. [22] captured Internet Gaming Disorder (IGD) in a longitudinal study of Spanish adolescents (mean age: 13.43 years); predictors of IGD were found to be lower Conscientiousness and lower Agreeableness.

In summary, the findings to date on associations between problematic gaming and the Big Five in minors are not consistent. The most frequent relations reported, were lower Conscientiousness, followed by lower Agreeableness and (somewhat less frequently) higher levels of Neuroticism. There are fewer empirical findings on other adolescent problematic behavioral patterns (e.g., use of social media and consumption of alcohol). Wang et al. [17] assessed “Social networking addiction” (a subtype of problematic social media use) and observed relationships with higher Extraversion and more pronounced Neuroticism in Chinese adolescents (average age: 15.03 years). Sánchez-Llorens et al. [20] reported statistically significant correlations between problematic alcohol use and higher Neuroticism, whereas Ibáñez et al. [23] found associations with lower Conscientiousness and Agreeableness. Surprisingly, findings on associations between problematic gaming and the facets of the Big Five (e.g., according to NEO-PI-R or BFI-2) are completely lacking so far. Thereby, according to Soto and John [15] findings capture “…facet-level traits meaningful personality information…” and “…individual facets relate uniquely with important behaviors and life outcomes” (p. 118). In addition, as far as we know, a comparison of the associations of the Big Five with substance-related and non-substance-related problematic behavioral patterns has not yet been conducted. To fill these research gaps, the present evaluation, in addition to determining the associations to the facets of the Big Five for the first time, we will additionally compare the associations between problematic gaming, problematic social media use, and problematic alcohol use in adolescence. For the present study, the following research questions were derived from this:

Research question 1: What are the empirical relationships between problematic gaming and the Big Five personality domains and their 15 facets in adolescence?

Research question 2: What are the empirical relationships between problematic social media use and the Big Five personality domains and their 15 facets in adolescence?

Research question 3: What are the empirical relationships between problematic alcohol use and the Big Five personality domains and their 15 facets in adolescence?

Research question 4: Are there differences in the empirical relationships of the Big Five personality domains and their 15 facets between problematic gaming, problematic social media use and problematic alcohol use in youth?

## 2. Materials and Methods

### 2.1. Procedure

Between January to March 2020, an established market research institute collected the data for the 5th wave of the VEIF project (for which cross-sectional statistical analyses are presented below). The Big Five personality domains and their 15 facets were surveyed in this wave in the sample of the longitudinal VEIF project for the first time (for the investigation of personality dimensions, a minimum age of 16 years is often recommended in the scientific literature, which the adolescents had, on average, just reached in this wave of the longitudinal survey). The study was approved by the Ethics Committee of the German Educational Research Association (Deutsche Gesellschaft für Erziehungswissenschaft, DGfE, approval number: 01/2018/DGfE). The data collection was conducted by 82 interviewers directly at the families’ homes. In each family, one adolescent and one associated parent should be explored (dyadic research approach, but only the youth were questioned about the Big Five personality domains and their 15 facets). Altogether, 491 family dyads and one adolescent (without parent) could be surveyed. In the VEIF project, a sample with an increased risk of problematic use of digital media compared to the general population was investigated. For this purpose, before the first collection of data was started in 2016, an oversampling of youth with an increased risk of problematic use of digital media was realized (a more detailed description of the study design and the recruitment process conducted at the outset of this research project can be found in [24]).

### 2.2. Measures

Problematic gaming in the last 12 months was surveyed in adolescent self-judgment using the Internet Gaming Disorder Scale (IGDS, [25]). The IGDS consists of nine items with a binary response format (0 = ‘no’, 1 = ‘yes’). A sum value (range: 0 to 9) can be calculated from the answers to the nine questions of the IGDS, and a higher sum value indicates more pronounced problematic gaming. The reliability coefficient of the IGDS in the sample studied was 0.83.

To measure problematic social media use in self-judgment in the past 12 months, the Social Media Disorder Scale (SMDS, [8]) was utilized. The SMDS also comprises nine questions with a binary response format (0 = ‘no’, 1 = ‘yes’). A total value (range: 0 to 9) can be calculated for the nine items of the SMDS. A higher sum value indicates more pronounced problematic social media use. The reliability coefficient of the SMDS in the surveyed sample was 0.80.

Problematic alcohol use in the last 12 months was collected with the Alcohol Use Disorders Identification Test-Consumption (AUDIT-C, [26]) that is recommended as a screening tool for adolescents [27]. The AUDIT-C consists of a total of three items with an alternating five-point response format. In the first question, the youth were asked how often they had a drink containing alcohol (five-level response format: 0 = ‘never’, 1 = ‘monthly or less’, 2 = ‘2 to 4 times a month’, 3 = ‘2 to 3 times a week’, 4 = ‘4 or more times a week’). In the second question, the adolescents should rate how many drinks containing alcohol they consume on a typical day when they are drinking (five-step response format: 0 = ‘1 to 2’, 1 = ‘3 to 4’, 2 = ‘5 to 6’, 3 = ‘7 to 9’, 4 = ‘10 or more’). In the third question, the youth were surveyed how often they consume six or more drinks on one occasion (five-step response format: 0 = ‘never’, 1 = ‘less than monthly’, 2 = ‘monthly’, 3 = ‘weekly’, 4 = ‘daily or almost daily’). A sum score (range: 0 to 12) can be calculated from the responses to the three items of the AUDIT-C, and a higher score indicates more pronounced problematic alcohol consumption. The reliability coefficient of the AUDIT-C in the sample investigated was McDonald’s ω = 0.66.

The Big Five personality traits and their 15 facets were surveyed with the established Big Five Inventory-2 (BFI-2, [15]), for this purpose the German version of the BFI-2 by Danner et al. [28] was applied. The reliability coefficients (McDonald’s omega) for the Big Five domains (according to BFI-2) in the sample studied were: (I) Open-Mindedness: ω = 0.82, (II) Conscientiousness: ω = 0.89, (III) Extraversion: ω = 0.80, (IV) Agreeableness: ω = 0.85 and for (V) Negative Emotionality: ω = 0.81. The reliability coefficients (McDonald’s omega) for the three facets of Open-Mindedness were: (A) Aesthetic Sensitivity: ω = 0.74, (B) Intellectual Curiosity: ω = 0.62 and (C) Creative Imagination: ω = 0.65. The reliability coefficients for the three facets of Conscientiousness were: (D) Organization: ω = 0.81, (E) Productiveness: ω = 0.75, as well as (F) Responsibility: ω = 0.70. The reliability coefficients for the three facets of Extraversion were: (G) Sociability: ω = 0.71, (H) Assertiveness: ω = 0.67 and (I) Energy Level: ω = 0.48. The reliability coefficients for the three facets of Agreeableness were: (J) Compassion: ω = 0.70, (K) Respectfulness: ω = 0.75, as well as (L) Trust: ω = 0.56. The reliability coefficients for the three facets of Negative Emotionality were: (M) Anxiety: ω = 0.51, (N) Depression: ω = 0.72 and (O) Emotional Volatility: ω = 0.64.

### 2.3. Sample Description

The sample comprises 492 adolescents (217 girls or 44.1% of the total sample and 275 boys or 55.9%). The average age of the youth was 16.83 years (SD = 0.97). A total of 379 of the adolescents surveyed were still in school and the other 113 had finished their school careers. Of these 113 cases, 20 (4.1% of the total sample) had graduated from school at a low education level, 80 (16.3%) at a medium education level and 12 (2.4%) at a high education level. Another person (0.2%) had left school without graduating. For the remaining 379 youth who were still attending school, parents were asked to predict which school diploma their children were likely to achieve based on current performance. Parents predicted graduation at a low education level for 25 adolescents (5.1% of the total sample), at a medium education level for another 158 (32.1%), and at a high education level for a total of 196 persons (39.8%).

### 2.4. Statistical Analyses

The statistical software SPSS (version 25.0, IBM, 2017, New York, NY, USA) was used to calculate frequencies, means, standard deviations, reliability coefficients (McDonald’s omega as recommended by Hayes and Coutts [29]), correlation analyses and multiple linear regression analyses. The dependent variables in the cross-sectional multiple linear regression analyses were: (A) problematic gaming, (B) problematic social media use and (C) problematic alcohol use. The explanatory variables were the same for all multivariable analyses each: first, gender, age and the Big Five personality domains (Open-Mindedness, Conscientiousness, Extraversion, Agreeableness and Negative Emotionality), as well as afterwards, gender, age and the 15 personality facets (Aesthetic Sensitivity, Intellectual Curiosity, Creative Imagination, Organization, Productiveness, Responsibility, Sociability, Assertiveness, Energy Level, Compassion, Respectfulness, Trust, Anxiety, Depression and Emotional Volatility).

## 3. Results

### 3.1. Bivariate Relationships between Problematic Behavioral Patterns and the Big Five Personality Domains

Correlation analyses revealed statistically significant bivariate associations between problematic gaming and male gender and all Big Five personality domains (lower Open-Mindedness, lower Conscientiousness, lower Extraversion, lower Agreeableness and higher Negative Emotionality, see Table 1). For problematic social media use, bivariate relations to lower age and three of the Big Five personality domains (lower Conscientiousness, lower Agreeableness and higher Negative Emotionality, see also Table 1) were observed. Problematic alcohol use was associated with male gender, higher age, as well as four of the Big Five personality domains (lower Open-Mindedness, lower Conscientiousness, lower Agreeableness and higher Negative Emotionality, see also Table 1).

### 3.2. Multivariable Relationships between Problematic Behavioral Patterns and the Big Five Personality Domains

In the multiple linear regression analysis with all Big Five personality domains, we obtained statistically significant associations between problematic gaming and male gender as well as lower Open-Mindedness and higher Negative Emotionality (explained variance: Corrected *R*^2^ = 0.24, see Table 2). Problematic social media use was connected with higher Extraversion and higher Negative Emotionality (explained variance: Corrected *R*^2^ = 0.08, see also Table 2). For problematic alcohol use, we found relations to male sex, older age, lower Open-Mindedness, as well as higher Extraversion and higher Negative Emotionality (explained variance: Corrected *R*^2^ = 0.24, see right column of Table 2).

### 3.3. Bivariate Relationships between Problematic Behavioral Patterns and the 15 Personality Facets

In the correlation analyses, statistically significant bivariate associations were found between problematic gaming and male gender as well as 14 of the 15 personality facets according to the BFI-2 (lower Aesthetic Sensitivity, lower Intellectual Curiosity, lower Creative Imagination, lower Organization, lower Productiveness, lower Responsibility, lower Sociability, lower Energy Level, lower Compassion, lower Respectfulness, lower Trust, higher Anxiety, higher Depression and higher Emotional Volatility, see Table 3). For problematic social media use, bivariate relationships were shown with lower age and with 12 of the 15 personality facets (lower Intellectual Curiosity, lower Creative Imagination, lower Organization, lower Productiveness, lower Responsibility, lower Energy Level, lower Compassion, lower Respectfulness, lower Trust, higher Anxiety, higher Depression and higher Emotional Volatility, see also Table 3). Problematic alcohol use was related to male gender and higher age as well as 10 out of 15 personality facets (lower Aesthetic Sensitivity, lower Creative Imagination, lower Organization, lower Productiveness, lower Responsibility, lower Energy Level, lower Compassion, lower Respectfulness, higher Depression and higher Emotional Volatility, see also Table 3).

### 3.4. Multivariable Relationships between Problematic Behavioral Patterns and the 15 Personality Facets

In the multiple linear regression analysis with the 15 facets of the BFI-2, statistically significant associations were found between problematic gaming and male gender as well as lower Aesthetic Sensitivity (facet of Open-Mindedness), higher Organization and lower Productiveness (facets of Conscientiousness), higher Assertiveness (facet of Extraversion) and higher Anxiety (facet of Negative Emotionality (explained variance in this multivariable regression model: Corrected *R*^2^ = 0.26, see Table 4)). Problematic social media use was related to higher Anxiety (facet of Negative Emotionality, explained variance: Corrected *R*^2^ = 0.09, see also Table 4) at the facet level. For problematic alcohol use, statistically significant relationships were observed with male gender, older age and higher Assertiveness (facet of Extraversion (explained variance: Corrected *R*^2^ = 0.24, see right column of Table 4)).

## 4. Discussion

In the present study, for the first time for adolescence, an investigation of the associations between problematic gaming, problematic social media use, problematic alcohol use and the facets of the Big Five was conducted (based on an established instrument, the Big Five Inventory-2, BFI-2 [15]). The examination of facets of the Big Five, not only of the personality domains themselves, offers a further differentiation which can bring additional knowledge [15]. Furthermore, a comparison should be made regarding the associations of both Big Five domains and their facets with various problematic behavioral patterns (problematic gaming, problematic social media use and problematic alcohol use) in youth.

In the following section, the empirical findings will be discussed in more detail. In particular, we will assess and interpret the results that were consistently found in the bivariate analyses as well as in the multivariable analyses and thus seem to us to be better validated. At the level of the Big Five personality domains, bivariate and multivariate relations between problematic gaming and lower Open-Mindedness and higher Negative Emotionality emerged consistently. For problematic alcohol use, associations with lower Open-Mindedness and higher Negative Emotionality were also observed, and also for problematic social media use there was an empirical relationship with higher Negative Emotionality.

According to these findings, in adolescence a higher Negative Emotionality or more pronounced Neuroticism (as the construct is referred to in other instruments for the five-factor model or for the assessment of the Big Five) seems to be uniformly relevant for both substance-related (problematic alcohol use) and non-substance-related behaviors (problematic gaming and problematic social media use). These results on Negative Emotionality correspond well with published findings concerning problematic alcohol use [20] and problematic social media use [17]. In contrast, problematic gaming showed associations with higher Neuroticism only in some surveys in adolescence (e.g., [19]), but not in other studies (e.g., [20]). From a cross-age perspective, the importance of Neuroticism for problematic gaming seems clearer; for example, the authors of two systematic reviews [30,31], in which findings for adults and adolescents are reported together, emphasize the unity of the relationships in their overviews. Şalvarlı and Griffiths [31] expressly emphasize that “no studies found a relationship between low neuroticism and IGD [Internet gaming disorder]” (p. 1436); such consistency was not empirically shown for all dimensions of the Big Five. Neuroticism is generally characterized by heightened negative experience and gaming could be used as a coping strategy against it or according to Şalvarlı and Griffiths [31] be interpreted “…as a way of preventing or inhibiting negative feelings…” (p. 1436). 

An addition to the body of research provided by this study, are the subsequent analyses at the facet level of the Big Five. Of the three facets of Negative Emotionality, the importance of stronger Anxiety was observed consistently in bivariate and multivariate analyses for both problematic gaming and problematic social media use. According to Soto and John [15], the facet Anxiety generally refers to “…the tendency to experience anxiety and fear…” (p. 122). The contents of the four items of facet Anxiety of the German version of the BFI-2 [28] relate to the experience of tension, nervousness and worry. To counteract this unpleasant experience, video games or social media (e.g., for distraction or to gain more positive emotions in the short term) could be used. With a higher expression of the facet Anxiety, the use of video games or social media could be correspondingly stronger or more excessive. Şalvarlı and Griffiths [31] mention “escaping their problems” (p. 1436) as an additional possible motive for problematic video game use for those affected.

As another Big Five personality domain, lower Open-Mindedness was statistically significantly associated with problematic gaming and problematic alcohol use in both bivariate analyses and multivariate evaluations. Relations between lower Open-Mindedness and problematic gaming had rarely been shown in adolescence, in the studies published to date (e.g., [17]), and there were no such relationships in most published investigations. In the two systematic reviews mentioned above [30,31], which summarized the state of research for adolescents and adults, heterogeneous findings were observed (roughly a similar number of studies showed that there were no statistically significant findings or that problematic gaming was associated with a lower level of Open-Mindedness). Open-Mindedness was thus somewhat more frequently related to problematic gaming in adulthood than in adolescence. With regard to the present survey, it should be noted that the youth, with an average age of almost 17 years, were a few years older than those affected in most published findings with adolescent samples (e.g., [16]). This age difference could possibly be an explanatory approach for the deviations from the available, published findings for youth. Clearly, more research is needed on the Big Five personality domain Open-Mindedness to better assess the strength and relevance of the associations to non-substance-related and substance-related problematic behaviors. An interesting aspect on the role of Open-Mindedness, which might also be relevant for the present study, can be found in Gervasi et al. [30] according to which “…gamers with low openness to experience tend to stick to their gaming behavior instead of exploring new activities, thus making low openness to experience even more important for the maintenance of IGD than for the onset of IGD” (p. 303).

This idea of a strong focus on problematic gaming and little interest in other activities also fits well with the findings on the facet-level for Open-Mindedness. Of the three facets of Open-Mindedness, a connection of problematic gaming to lower pronounced Aesthetic Sensitivity was shown in both bi- and multivariate analyses. The items of the BFI-2 record for facet Aesthetic Sensitivity, the general interest in art and music, plays or literature (e.g., poems) are mentioned as exemplary contents. Problematic gaming often leads to a narrowing of interests, and it is quite conceivable that other forms of art (such as stage plays) may seem much less attractive to youth, who show little interest in engaging with them (especially if they are presented offline). However, for some of the types of art mentioned (e.g., poetry), intensive engagement with it might be an important prerequisite for minors to gain access to it or develop an interest in it (which might be difficult to reconcile with a time-intensive hobby such as gaming).

Only at the level of the facets but not at the level of the personality domains, statistically significant bivariate and multivariate relations between problematic gaming and lower Productiveness (a facet of Conscientiousness) were found (see Table 3 and Table 4). Productiveness is generally understood to mean a “…work ethic and persistence while pursuing goals…”, which “…helps explain why this domain is a powerful predictor of academic achievement and job performance” according to Soto and John [15]. The contents of the four items of the facet Productiveness of the BFI-2 survey persistence and efficiency in completing tasks or laziness and postponing tasks. Problematic gaming, in addition to a focus on this behavior (as already named above), is often accompanied by a large amount of time spent on this behavior (for example, to complete tasks in the game). This large time commitment to gaming is permanently difficult to reconcile with high commitment or special diligence in other areas of life (e.g., in the context of school or vocational training), so the associations with lower Productiveness seem quite plausible. This interesting finding on the importance of lower Productiveness for problematic gaming extends the state of the art in research, but obviously needs replications in further examinations. In this context, it should be checked in further samples whether the associations to lower Conscientiousness more frequently reported in the literature (e.g., [19]), which in the present study only showed up in the bivariate but no longer in the multivariable statistical analysis, can really be explained primarily by associations to lower Productiveness or whether the other facets of Conscientiousness (Organization and Responsibility) are also relevant.

The present study offers additional insights beyond the state of the research (particularly at the facet level of the Big Five) but has several limitations. The results were collected in a sample of older adolescents; but to what extent these findings are transferable to adulthood cannot be answered. In this survey, no representative sample was examined and compared to the general population, youth with subjectively perceived problems in digital media use were deliberately oversampled (for further details on the sampling strategy see Wartberg et al. [24]). Furthermore, in problematic digital media use in adolescence, in addition to self-ratings, there is the approach of working with external assessments (often parental judgments, see, e.g., [32]). External ratings are also used in the measurement of personality traits (e.g., in the validation of the BFI-2 by Soto and John [15]), but in the present study this additional source of information was not incorporated. With regard to reliability, Aiken and Groth-Marnat [33] recommended 0.60 as the limit for the reliability of an instrument in group surveys. While at the level of the personality domains good values for the reliability coefficients (between 0.80 and 0.89, determined with McDonald’s omega) were observed, three of the 15 facets (Energy Level, Trust and Anxiety) showed clearly lower reliability (<0.60) in the investigated sample than recommended by Aiken and Groth-Marnat [33] and previously observed in the validation studies of the BFI-2 by Soto and John [15] or Danner et al. [28], which has to be taken into account when interpreting the results. One possible explanation for this is the age difference in the samples examined; while adolescents were interviewed in the present survey, both Soto and John [15] and Danner et al. [28] surveyed adults. As described, the five-factor model is the most widely used model of personality dimensions in research. However, there are other approaches to capture personality beyond this, e.g., the concept of Sensation Seeking. Initial studies have examined associations of problematic gaming and sensation seeking, but the findings are not consistent [34,35]. Alternative approaches to assessing personality dimensions, in addition to the Big Five, were not considered in the present investigation. Several other constructs have been empirically associated with excessive use of digital media; in particular, problems in emotion regulation are rated as relevant for different problematic behavioral patterns (e.g., [36]), but were not surveyed in this study.

## 5. Conclusions

The aim of this manuscript was to make a first empirical contribution to explore etiological similarities and differences in personality dimensions of the Big Five and their facets between substance-related (problematic alcohol use) and non-substance-related problematic behavior patterns (problematic gaming or problematic social media use). For the investigated substance-related and non-substance-related problematic behaviors in adolescence, very similar associations to the Big Five emerged at both the personality domain and facet levels. Considering the confidence intervals, which often overlapped clearly in the present study, there was no difference in the associations between problematic gaming, problematic social media use, and problematic alcohol use with regard to the personality domain Open-Mindedness; for example, although statistically significant relationships were only found for two of the three problematic behavioral patterns (see Table 2). Confidence intervals that do not overlap for the three problematic behaviors show up for very few of the characteristics examined: (A) Male gender was related to problematic gaming but not to problematic social media use (see Table 2 and Table 4), (B) older age was associated with problematic alcohol consumption but not with problematic gaming or problematic social media use (see Table 2 and Table 4) and (C) the facet Anxiety was related to problematic gaming but not to problematic alcohol use (see Table 2). The finding that boys exhibit problematic gaming more often than girls (A) is well known from the literature (e.g., [37]). With regard to the findings on “older age” (B), it must be taken into account that in Germany certain alcoholic beverages can already be legally purchased from the age of 16 and thus in adolescence, compared to other countries, the legal age for alcohol consumption is thus rather low (e.g., [38]). Given the age range of the sample surveyed (15 to 19 years) the immediate relevance of an older age for purchasing and consuming alcohol becomes clear. The finding on the facet Anxiety (C) extended, as far as we know, the previous state of research. In contrast, for all five personality domains of the Big Five and for the 14 other facets (besides Anxiety), the confidence intervals overlap, indicating that the relationships to problematic gaming, problematic social media use and problematic alcohol use did not differ.

With respect to personality (both Big Five personality domains and associated facets), these empirical findings can be interpreted as preliminary evidence for a similar etiology of substance-related and non-substance-related problematic behaviors in youth. This evidence of comparable etiology for non-substance-related and substance-related behaviors did not show such uniformity for other characteristics. For example, both increased hyperactivity/inattention (aspect of psychopathology, see [39]) and decreased autonomy (aspect of parent–child relationship, see [40]) among adolescents were statistically significantly associated with problematic gaming and problematic social media use but not with problematic alcohol use (no overlap of respective confidence intervals). In the study by Estévez et al. [41], it was found that in a sample of adolescents and young adults, attachment (in this case peer attachment) was related to problematic gaming, but not to problematic alcohol use. With regard to the personality dimensions of the Big Five including the facets, on the other hand, differences between substance-related and non-substance-related problematic behavioral patterns in adolescents hardly seem to be present. But of course, the findings of the present study require replications in the cross-section and additional validation as to what extent they also show up in the longitudinal section.

## Figures and Tables

**Table 1:** Correlation matrix for the problematic behavioral patterns and the Big Five personality domains (*N* = 492).

Variable	1	2	3	4	5	6	7	8	9	10
(1) Internet gaming disorder	–									
(2) Problematic social media use	0.44 ***	–								
(3) Problematic alcohol use	0.13 **	0.07	–							
(4) Gender ^a^	−0.35 ***	0.04	−0.17 ***	–						
(5) Age	−0.07	−0.09 *	0.42 ***	−0.00	–					
(6) Open-Mindedness (BFI-2)	−0.27 ***	−0.09	−0.14 **	0.24 ***	0.02	–				
(7) Conscientiousness (BFI-2)	−0.34 ***	−0.19 ***	−0.17 ***	0.25 ***	0.05	0.48 ***	–			
(8) Extraversion (BFI-2)	−0.16 ***	−0.06	−0.00	0.05	−0.01	0.38 ***	0.39 ***	–		
(9) Agreeableness (BFI-2)	−0.31 ***	−0.19 ***	−0.15 ***	0.23 ***	0.03	0.21 ***	0.65 ***	0.24 ***	–	
(10) Negative Emotionality (BFI-2)	0.30 ***	0.28 ***	0.09 *	0.04	−0.10 *	−0.15 ***	−0.56 ***	−0.45 ***	−0.60 ***	–

Note. ^a^ Coding: 0 = male, 1 = female. *** *p* < 0.001; ** *p* < 0.01; * *p* < 0.05.

**Table 2:** Associations between the Big Five domains and problematic gaming, problematic social media use and problematic alcohol use in the multivariable regression analyses (*N* = 492).

Variable	Internet Gaming Disorder Standardized Beta Coefficients (95% CI)	Problematic Social Media Use Standardized Beta Coefficients (95% CI)	Problematic Alcohol Use Standardized Beta Coefficients (95% CI)
Gender ^a^	−0.31 *** (−0.39; −0.22)	0.05 (−0.04; 0.14)	−0.13 ** (−0.22; −0.05)
Age	−0.04 (−0.11; 0.04)	−0.06 (−0.14; 0.03)	0.44 *** (0.37; 0.52)
Open-Mindedness (BFI-2)	−0.14 ** (−0.24; −0.05)	−0.08 (−0.18; 0.03)	−0.12 * (−0.21; −0.02)
Conscientiousness (BFI-2)	−0.04 (−0.16; 0.08)	−0.03 (−0.17; 0.10)	−0.07 (−0.20; 0.05)
Extraversion (BFI-2)	0.06 (−0.04; 0.15)	0.11 * (0.00; 0.21)	0.15 ** (0.05; 0.24)
Agreeableness (BFI-2)	−0.03 (−0.15; 0.08)	−0.02 (−0.14; 0.10)	−0.01 (−0.12; 0.10)
Negative Emotionality (BFI-2)	0.27 *** (0.16; 0.39)	0.28 *** (0.15; 0.40)	0.14 * (0.03; 0.25)
Corrected *R*^2^	0.24	0.08	0.24

Note. ^a^ Coding: 0 = male, 1 = female. *** *p* < 0.001; ** *p* < 0.01; * *p* < 0.05.

**Table 3:** Correlation matrix for the problematic behavioral patterns and the 15 personality facets (*N* = 492).

Variable	1	2	3	4	5	6	7	8	9	10	11	12	13	14	15	16	17	18	19	20
(1) Internet gaming disorder	–																			
(2) Problematic social media use	0.44 ***	–																		
(3) Problematic alcohol use	0.13 **	0.07	–																	
(4) Gender ^a^	−0.35 ***	0.04	−0.17 ***	–																
(5) Age	−0.07	−0.09 *	0.42 ***	−0.00	–															
(6) Aesthetic Sensitivity ^b^	−0.24 ***	0.00	−0.15 ***	0.34 ***	−0.01	–														
(7) Intellectual Curiosity ^b^	−0.22 ***	−0.14 **	−0.08	0.14 **	0.07	0.57 ***	–													
(8) Creative Imagination ^b^	−0.18 ***	−0.09 *	−0.11 *	0.07	−0.01	0.37 ***	0.52 ***	–												
(9) Organization ^c^	−0.25 ***	−0.09 *	−0.18 ***	0.25 ***	0.01	0.37 ***	0.41 ***	0.34 ***	–											
(10) Productiveness ^c^	−0.34 ***	−0.18 ***	−0.10 *	0.22 ***	0.09 *	0.32 ***	0.46 ***	0.40 ***	0.70 ***	–										
(11) Responsibility ^c^	−0.32 ***	−0.23 ***	−0.16 ***	0.18 ***	0.04	0.14 **	0.34 ***	0.32 ***	0.58 ***	0.68 ***	–									
(12) Sociability ^d^	−0.16 ***	−0.07	0.01	0.05	0.04	−0.07	0.16 ***	0.30 ***	0.23 ***	0.29 ***	0.33 ***	–								
(13) Assertiveness ^d^	−0.00	0.01	0.07	−0.09 *	−0.03	0.15 **	0.29 ***	0.47 ***	0.11 *	0.24 ***	0.10 *	0.46 ***	–							
(14) Energy Level ^d^	−0.24 ***	−0.10 *	−0.10 *	0.18 ***	−0.04	0.26 ***	0.42 ***	0.46 ***	0.36 ***	−0.45 ***	0.44 ***	0.50 ***	0.46 ***	–						
(15) Compassion ^e^	−0.28 ***	−0.15 **	−0.16 ***	0.25 ***	0.00	0.10 *	0.27 ***	0.30 ***	0.46 ***	0.43 ***	0.64 ***	0.26 ***	0.01	0.41 ***	–					
(16) Respectfulness ^e^	−0.32 ***	−0.21 ***	−0.14 **	0.19 ***	−0.01	0.08	0.28 ***	0.23 ***	0.53 ***	0.50 ***	0.68 ***	0.24 ***	−0.01	0.37 ***	0.71 ***	–				
(17) Trust ^e^	−0.19 ***	−0.11 *	−0.08	0.14 **	0.08	−0.05	0.06	0.06	0.30 ***	0.34 ***	0.51 ***	0.21 ***	−0.15 **	0.18 ***	0.54 ***	0.50 ***	–			
(18) Anxiety ^f^	0.27 ***	0.27 ***	0.04	0.09 *	−0.09 *	0.14 **	−0.10 *	−0.21 ***	−0.21 ***	−0.32 ***	−0.42 ***	−0.40 ***	−0.17 ***	−0.27 ***	−0.30 ***	−0.37 ***	−0.36 ***	–		
(19) Depression ^f^	0.26 ***	0.21 ***	0.10 *	0.00	−0.04	0.05	−0.24 ***	−0.34 ***	−0.33 ***	−0.40 ***	−0.58 ***	−0.54 ***	−0.33 ***	−0.50 ***	−0.43 ***	−0.51 ***	−0.38 ***	0.62 ***	–	
(20) Emotional Volatility ^f^	0.24 ***	0.23 ***	0.10 *	0.01	−0.12 **	0.02	−0.21 ***	−0.18 ***	−0.44 ***	−0.47 ***	−0.59 ***	−0.25 ***	−0.02	−0.27 ***	−0.45 ***	−0.58 ***	−0.45 ***	0.55 ***	0.53 ***	–

Note. ^a^ Coding: 0 = male, 1 = female. ^b^ Facet of Open-Mindedness (BFI-2). ^c^ Facet of Conscientiousness (BFI-2). ^d^ Facet of Extraversion (BFI-2). ^e^ Facet of Agreeableness (BFI-2). ^f^ Facet of Negative Emotionality (BFI-2). *** *p* < 0.001; ** *p* < 0.01; * *p* < 0.05.

**Table 4:** Associations between the Big Five facets and problematic gaming, problematic social media use and problematic alcohol use in the multivariable regression analyses (*N* = 492).

Variable	Internet Gaming Disorder Standardized Beta Coefficients (95% CI)	Problematic Social Media Use Standardized Beta Coefficients (95% CI)	Problematic Alcohol Use Standardized Beta Coefficients (95% CI)
Gender ^a^	−0.27 *** (−0.36; −0.19)	0.04 (−0.05; 0.14)	−0.11 * (−0.19; −0.02)
Age	−0.04 (−0.11; 0.04)	−0.06 (−0.14; 0.03)	0.43 *** (0.35; 0.51)
Aesthetic Sensitivity ^b^	−0.16 ** (−0.27; −0.05)	0.01 (−0.11; 0.13)	−0.10 (−0.21; 0.01)
Intellectual Curiosity ^b^	−0.01 (−0.12; 0.10)	−0.09 (−0.21; 0.03)	0.02 (−0.09; 0.13)
Creative Imagination ^b^	−0.02 (−0.12; 0.08)	−0.01 (−0.13; 0.11)	−0.08 (−0.18; 0.02)
Organization ^c^	0.13 * (0.01; 0.25)	0.13 (−0.00; 0.26)	−0.09 (−0.21; 0.03)
Productiveness ^c^	−0.17 * (−0.30; −0.04)	−0.09 (−0.24; 0.06)	0.06 (−0.08; 0.19)
Responsibility ^c^	−0.01 (−0.15; 0.13)	−0.11 (−0.26; 0.04)	−0.07 (−0.21; 0.07)
Sociability ^d^	−0.05 (−0.15; 0.06)	0.05 (−0.06; 0.17)	0.04 (−0.06; 0.15)
Assertiveness ^d^	0.13 * (0.02; 0.23)	0.09 (−0.03; 0.21)	0.15 ** (0.05; 0.26)
Energy Level ^d^	−0.01 (−0.12; 0.10)	0.00 (−0.12; 0.12)	−0.01 (−0.12; 0.10)
Compassion ^e^	−0.00 (−0.09; 0.09)	0.02 (−0.11; 0.16)	−0.04 (−0.17; 0.08)
Respectfulness ^e^	−0.13 (−0.26; 0.00)	−0.10 (−0.25; 0.04)	0.09 (−0.04; 0.23)
Trust ^e^	0.07 (−0.03; 0.17)	0.08 (−0.04; 0.19)	0.02 (−0.08; 0.11)
Anxiety ^f^	0.22 *** (0.12; 0.33)	0.20 ** (0.08; 0.32)	0.01 (−0.09; 0.12)
Depression ^f^	0.06 (−0.07; 0.18)	0.04 (−0.10; 0.17)	0.11 (−0.02; 0.23)
Emotional Volatility ^f^	−0.00 (−0.09; 0.09)	0.02 (−0.11; 0.15)	0.07 (−0.04; 0.19)
Corrected *R*^2^	0.26	0.09	0.24

Note. ^a^ Coding: 0 = male, 1 = female. ^b^ Facet of Open-Mindedness (BFI-2). ^c^ Facet of Conscientiousness (BFI-2). ^d^ Facet of Extraversion (BFI-2). ^e^ Facet of Agreeableness (BFI-2). ^f^ Facet of Negative Emotionality (BFI-2). *** *p* < 0.001; ** *p* < 0.01; * *p* < 0.05.

## Data Availability

The data presented in this study are available on reasonable request from the corresponding author.
